# Establishment and characterization of a new hypoxia-resistant cancer cell line, OCUM-12/Hypo, derived from a scirrhous gastric carcinoma

**DOI:** 10.1038/sj.bjc.6605543

**Published:** 2010-02-09

**Authors:** Y Kato, M Yashiro, S Noda, M Tendo, S Kashiwagi, Y Doi, T Nishii, J Matsuoka, Y Fuyuhiro, O Shinto, T Sawada, M Ohira, K Hirakawa

**Affiliations:** 1 Department of Surgical Oncology, Osaka City University Graduate School of Medicine, 1-4-3 Asahi-machi, Abeno-ku, Osaka, Japan; 2 Oncology Institute of Geriatrics and Medical Science, Osaka City University Graduate School of Medicine, 1-4-3 Asahi-machi, Abeno-ku, Osaka, Japan

**Keywords:** hypoxia resistant, scirrhous gastric carcinoma, cell line, epithelial-to-mesenchymal transition

## Abstract

**Background:**

Many kinds of solid tumour have heterogeneously a hypoxic environment. Tumour hypoxia reported to be associated with more aggressive tumour phenotypes such as high metastatic ability and resistance to various anti-cancer therapies which may lead to a poorer prognosis. However, the mechanisms by which hypoxia affects the aggressive phenotypes remain unclear.

**Methods:**

We established a scirrhous gastric carcinoma cell line (OCUM-12) from ascites associated with scirrhous gastric carcinoma, and a hypoxia-resistant cancer cell line (OCUM-12/Hypo) was cloned from OCUM-12 cells by continuous exposure to 1% oxygen.

**Results:**

Histologic findings from orthotopic tumours derived from parent OCUM-12 cells and daughter OCUM-12/Hypo cells revealed poorly differentiated adenocarcinoma with extensive fibrosis that resembled human scirrhous gastric cancer. Necrotic lesions were frequently detected in the OCUM-12 tumours but were rarely found in the OCUM-12/Hypo tumours, although both types had multiple hypoxic loci. Apoptosis rate of OCUM-12 cells was increased to 24.7% at 1% O_2_, whereas that of OCUM-12/Hypo was 5.6%. The OCUM-12/Hypo orthotopic models developed multiple metastases to the peritoneum and lymph nodes, but the OCUM-12 models did not. OCUM-12/Hypo cells showed epithelial-to-mesenchymal transition and high migratory and invasive activities in comparison with OCUM-12 cells. The mRNA expression levels of both *E-cadherin* and zonula occludens *ZO-1* and *ZO-2* decreased in OCUM-12/Hypo cells, and that of *vimentin*, *Snail-1*, *Slug/Snail-2*, *Twist*, *ZEB-1*, *ZEB-2*, matrix metalloproteinase-1 (*MMP-1*), and *MMP-2* were increased in OCUM-12/Hypo cells.

**Conclusion:**

OCUM-12 and OCUM-12/Hypo may be useful for the elucidation of disease progression associated with scirrhous gastric cancer in the setting of chronic hypoxia.

Most solid tumours are hypovascular and possess microscopically heterogeneous hypoxic regions (Vaupel *et al*, 1987, 2003; Hockel *et al*, 1996; Driessen *et al*, 2006; Mayer *et al*, 2008). Cancer cells under hypoxic conditions are reported to show malignant features, such as high metastatic ability and resistance to various anti-cancer therapies (Harris and Shrieve, 1979; Ludwig, 1984; Driessen *et al*, 2006); however, the mechanisms by which hypoxia affects cancer progression remain unclear. Hypoxia alters important intracellular pathways, and it is recognized as a stimulus for the malignant potential (Semenza, 2002; Subarsky and Hill, 2003; Vaupel, 2004). The hypoxic condition is heterogeneous not only regarding location, but also regarding duration (such as acute or chronic hypoxia) (Hockel and Vaupel, 2001). Although the effect of acute hypoxia on the phenotype of cancer cells has been often studied (Brown, 1979), few reports of chronic hypoxia have been published (Song *et al*, 2001; Kim *et al*, 2009a). Cancer cells may frequently be exposed to continuous hypoxia because of their hypovascular nature. A hypoxia-resistant cell line might be useful for the study of cancer phenotypes under conditions of chronic hypoxia, however, to the best of our knowledge, so far there has been no report of the establishment of a hypoxia-resistant cell line. We established a new hypoxia-resistant cancer cell line derived from scirrhous gastric carcinoma, designated as OUCM-12/Hypo, that continued to grow in 1% oxygen. Human scirrhous gastric carcinoma (also known as linitis plastica carcinoma) is a diffuse-type gastric cancer characterized by cancer cell infiltration and proliferation and accompanied by extensive stromal fibrosis (Tahara, 1990). It may grow in hypoxic conditions due to a lesser degree of angiogenesis in comparison to intestinal-type carcinoma (Takahashi *et al*, 1996). Characterization of hypoxia-resistant cancer cells might elucidate the behaviour of scirrhous gastric carcinoma in hypoxic conditions.

## Materials and methods

### Patients

**Figure 1:**
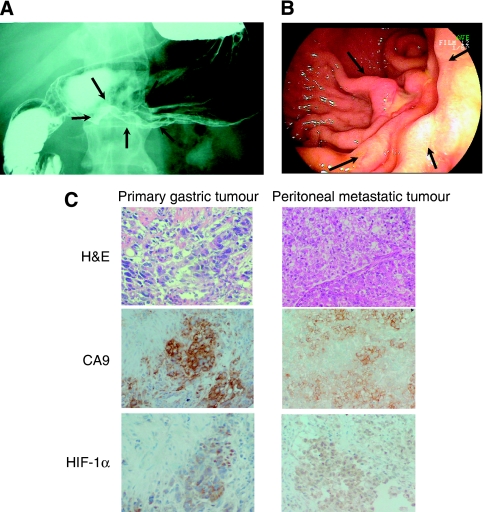
Upper gastrointestinal series (**A**) and gastro-fibrscopy (**B**) showed diffusely infiltrating carcinomas in which ulceration is usually not a marked feature (arrows). Histology of the primary tumour and the peritoneal metastatic tumour showed poorly differentiated adenocarcinoma (**C**). Primary gastric tumour accompanied by fibrosis. Immunostaining of hypoxia-inducible factor-1*α* (HIF-1*α*) and carbonic anhydrase 9 (CA9) showed that hypoxic lesions were present in both the primary tumour and the peritoneal metastatic tumour (**C**).

OCUM-12 was derived from ascites of a 58-year-old man with peritoneal dissemination of gastric carcinoma in 2004. Upper gastrointestinal series (Figure 1A) and gastroendoscopic examination (Figure 1B) found diffusely infiltrating gastric carcinomas in which ulceration is usually not a marked feature (Japanese Gastric Cancer Association, 1998). Examination of the biopsy specimen from primary gastric tumour revealed that it has the characteristic morphology of poorly differentiated adenocarcinoma accompanied by stromal cells (Figure 1C). Therefore, the primary tumour was diagnosed as scirrhous types of gastric carcinoma (Japanese Gastric Cancer Association, 1998). The tissue sections were subjected to immunohistochemical staining using antibodies against carbonic anhydrase 9 (CA9; 1 : 1000; Novus Biologicals, Littleton, CO, USA), and hypoxia-inducible factor-1*α* (HIF-1*α*; 1 : 25; Novus Biologicals). The staining of hypoxia-associated endogenous proteins, HIF-1*α* and CA9, was heterogeneously positive in cancer cells of both biopsy specimen and peritoneal metastasis (Figure 1C). Informed consent was obtained from the patients from whom the tumour specimens were obtained.

### Establishment of cell lines and cell culture

Abdominal effusion from a patient was collected aseptically into a bottle with heparin then centrifuged at 1000 r.p.m. for 5 min. The pellet was suspended in 10 ml culture medium (see below) and seeded into 100 mm culture dishes (Falcon, Lincoln Park, NJ, USA). Initial culture was performed in a humidified incubator at 37°C in an atmosphere of 5% carbon dioxide and 95% air. The culture medium was Dulbecco's modified Eagle's medium (DMEM; Wako, Osaka, Japan) with 10% heat-inactivated fetal bovine serum (FBS; Nichirei Biosciences, Tokyo, Japan), 100 IU ml^−1^ penicillin (Sigma, Steinheim, Germany), 100 *μ*g ml^−1^ streptomycin (Sigma), and 0.5 mM sodium pyruvate (Sigma). Floating cells and adherent mesothelial cells were found in a dish. Floating cells were collected and re-suspended in medium. Serial passages were carried out every 4–7 days. The cells were passaged routinely at a ratio of 1 : 5 or 1 : 10. The floating cell lines were designated OCUM-12. OCUM-12 cell was carried for more than 48 months and passaged for more than 200 generations. A new hypoxia-resistant cell line was established from OCUM-12, as follows: OCUM-12 cells at 180th passage generations were cultured in a humidified incubator at 37°C in an atmosphere of 5% carbon dioxide and 5% oxygen for 4 weeks. Although most of OCUM-12 cells were dead under hypoxic condition, some cancer cells were alive and grew gradually under 5% oxygen. Then, surviving cells were cultured for 6 weeks in 1% oxygen. Most cells were dead at initial culture, some cancer cells began to grow under 1% oxygen conditions. Proliferative cancer cells under 1% oxygen were designated OCUM-12/Hypo as a new hypoxia-resistant cell line. OCUM-12/Hypo cell was cultured for more than 5 months and passaged for more than 20 generations. Both cell lines were tested for *Mycoplasma* contamination with a Hoechst staining kit (Flow, Tokyo, Japan). Morphologic findings were investigated with a phase-contrast microscope. Cells were stained with hematoxylin and eosin (H&E). All experimental studies using OCUM-12 cells were carried out at 20% O_2_, and those using OCUM-12/Hypo cells were carried out at 1% O_2_.

### Growth kinetics

The doubling time of OCUM-12 and OCUM-12/Hypo cells was determined at the 190th and 10th passages, respectively. Briefly, suspensions of 1.0 × 10^4^ cells were incubated in 24-well dishes with 1 ml DMEM containing 10% FCS. Number of cancer cells was counted every 24 h by counting chamber. The doubling times were determined from the growth curve. Production of tumour-associated antigen was examined as follows: a suspension of 1.0 × 10^6^ cells in 100 mm plastic culture dishes was incubated for 3 days in culture medium. Then, production levels of tumour-associated antigens, carcinoembryonic antigen (CEA), carbohydrate 19-9 (CA19-9), SPan-1, and *α*-fetoprotein (AFP), in the spent medium were measured by radioimmunoassay as previously reported (Yashiro *et al*, 1995).

### Chromosome analysis

Cells were karyotyped using a standard air-dried method (Seabright, 1971) after treatment with a final concentration of 0.05 *μ*g ml^−1^ colcemid for 2 h when the cells were in an exponential growth phase. They were analysed using trypsin G banding. A total of 50 metaphase spreads were counted to determine the modal number. Karyotyping was performed according to the International System for Human Cytogenetic Nomenclature (Seabright, 1971). Chromosome analysis was carried out on the two cell lines at the 40th passage.

### DNA extraction

Genomic DNA of primary gastric tumour was extracted from the biopsy specimens after it had been fixed in 10% buffered formalin and embedded in paraffin. One section was stained with H&E and used as a reference for selecting areas for microdissection from adjacent sections, using a sterile scalpel blade under a dissecting microscope. Genomic DNA of OCUM-12 and OCUM-12/Hypo cells was extracted with phenol–chloroform. The genomic DNA was isolated from the paraffin-embedded microdomains removed from the sections using Proteinase K (Gibco Life Technologies, Gaithersburg, MD, USA) at a final concentration of 100 *μ*g ml^−1^ and was incubated for 5 h at 55°C.

### PCR-LOH/MSI assay

Microsatellite instability (MSI) and loss of heterozygosity (LOH) were analysed by PCR-LOH/MSI assay, as previously reported (Fujiwara *et al*, 2008). A 6 *μ*l aliquot of reaction mixture was formulated using 0.4 *μ*l of DNA extracted from normal epithelium, primary tumour, OCUM-12, and OCUM-12/Hypo cells. 4.5 *μ*l of each primer marker set, 0.06 *μ*l AmpliTaq Gold (Applied Biosystems, Foster City, CA, USA), and 1.04 *μ*l distilled water. Four primer marker sets – *TP53Penta*, *D18S35*, *D3S1611*, and *D5S346 –* were linked to the *p53* loci on 17p13, the *DCC/DPC4* locus on 18q21, the *hMLH1* locus on 3p23-21.3, and the *APC* locus on 5q21. The polymerase chain reaction (PCR) conditions were as follows: 95°C for 10 min followed by 45 cycles (96°C for 10 s, 55°C for 30 s, 70°C for 3 min), with a final elongation at 70°C for 30 min. Each sample was analysed by the ABI PRISM 310 Genetic Analyzer (Applied Biosystems). A tumour was determined as exhibiting LOH if there was absence or more than a 50% reduction in the peak height of one allele of the tumour sample compared to the normal epithelium allele.

### Orthotopic tumour models

Orthotopic implantation was performed as previously reported (Yashiro *et al*, 1996; Takemura *et al*, 2004). Four-week-old BALB/c nude mice (Oriental Kobo, Osaka, Japan) was used as xenograft recipient for human tumour fragments. OCUM-12 cells (1.0 × 10^7^) and OCUM-12/Hypo cells (1.0 × 10^7^) suspended in a volume of 100 *μ*l DMEM were inoculated orthotopically into five mice at each experiment. Under ether anaesthesia, a median abdominal incision was made in nude mice. The stomach was exposed carefully, and 1.0 × 10^7^ cells were inoculated at the gastric antrum using 30-gauge needles (Handaya, Saitama, Japan). Seven weeks after inoculation, the hypoxic cell marker pimonidazole hydrochloride (Hypoxyprove-1 kit; Chemicon, Temecula, CA, USA) was used to delineate the hypoxic site of the tumours due to its specific binding to cells in a partial oxygen tension <10 mm Hg, as previously reported (Furuta *et al*, 2008). Pimonidazole (60 mg kg^−1^) was injected to the mouse through tail vein injection 90 min before the animals were killed. Upon killing the animal, the subcutaneous tumours, the gastric tumour, and metastatic tumours were removed and fixed in 10% formalin for paraffin sections and stained with H&E and Masson's trichrome. The selected tissue sections were subjected to immunohistochemical staining using antibodies against pimonidazole (1 : 50, hypoxyprobe-1; Chemicon), CA9, and HIF-1*α*. Animal experiments were performed in compliance with the guidelines of the Osaka City University Ethical Committee.

### Wound-healing assays

The *in vitro* wound-healing ability was measured using the method of Borensztajn *et al* (2008) with some modifications. Gastric cancer cells were cultured in six-well plates. After the cells reached semi-confluence, a wound was created in the cell monolayer by a pipette tip. Cancer cells were cultured for additional 48 h at 37°C. Four scratched fields were randomly chosen and the number of migration cells was counted. The culture was performed in duplicate.

### Invasion assay

The *in vitro* invasiveness was measured using the method of Albini *et al* (1987) with some modifications. We used the chemotaxicell chambers with a 12 *μ*m pore membrane filter (Kubota, Osaka, Japan) coated with 50 *μ*g of Matrigel in a 24-well culture plate. OCUM-12 and OCUM-12/Hypo cells were re-suspended to a final concentration of 1.0 × 10^4^ cells per ml in DMEM with 10% FBS. After incubation for 72 h in an atmosphere of 1 and 20% oxygen, cancer cells on the upper surface of the membrane were removed by wiping. The membrane was stained with hematoxylin. Cancer cells that invaded through a filter coated with Matrigel to the lower membrane were manually counted under a microscope at × 200 magnification. Four randomly chosen fields were counted for each assay. The mean of six fields was calculated as the sample value. For each group, the culture was performed in triplicate.

### Reverse transcription–PCR (RT–PCR)

Total cellular RNA was extracted from OCUM-12 and OCUM-12/Hypo gastric cancer cell lines (each cell lines incubate 20 and 1% oxygen for 24 h) with Trizol (Life Technologies Inc.) according to the manufacturer’s protocol. Next, cDNAs were synthesized from 1 *μ*g of the RNA with a Moloney murine leukemia virus-reverse transcription kit (Life Technologies Inc.) using random hexamers. The cDNAs were amplified by PCR for 30 cycles with AmpliTaq Gold DNA polymerase (PerkinElmer Cetus, Norwalk, CN, USA) on a thermal cycler with each primer (Table 1). The PCR conditions were: pre-denaturation, 94°C for 10 min; denaturation, 94°C for 60 s; annealing, 65°C for 60 s; extension, 72°C for 60 s; and final incubation, 72°C for 10 min. Reverse transcription–PCR was performed twice.

### Gelatin zymography

The gelatinolytic activity of matrix metalloproteinases-2 (MMP-2) secreted in conditioned media was assayed by means of gelatin substrate gel electrophoresis. Conditioned media were harvested and the protein concentration was measured, followed by concentrating by precipitation with two volumes of absolute ethanol. Protein (20 *μ*g) was re-suspended with two-sample buffer without reducing agent (0.5 M Tris–HCl (pH 6.8), 10% SDS, 0.1% bromophenol blue, 10% glycerol) and then subjected to 7.5% SDS–PAGE gel containing 0.1% (w/v) gelatin without prior boiling. After electrophoresis at 4°C, the gel was washed with 2.5% Triton X-100 (v/v) for 30 min, and subsequently incubated in substrate buffer (50 mM Tris–HCl (pH 7.5), 1 mM ZnCl_2_, 5 mM CaCl_2_) at 37°C for 48–72 h. The gel was then stained with 0.5% (w/v) Coomassie blue. Proteolytic activities were detected by clear bands indicating the lysis of the substrate. Gelatin zymography was carried out for two times.

### Flow cytometry

Apoptosis was detected using flow cytometry by staining cells with Annexin V–FITC and propidium iodide (BD Pharmingen, San Diego, CA, USA) labeling. OCUM-12 and OCUM-12/Hypo cells were seeded at a density of 1.0 × 10^5^ cells per ml in 100-mm plates. Using an apoptosis kit, we stained cells with Annexin V–FITC and propidium iodide according to the manufacturers’ instructions, and immediately analysed using FACScan flow cytometry (Becton Dickinson, Mountain View, CA, USA).

### Statistical analysis

Data were analysed using Student's *t*-test. *P*-values less than 0.05 were considered statistically significant.

## Results

### Characterization of OCUM-12 and OCUM-12/Hypo cells

**Figure 2:**
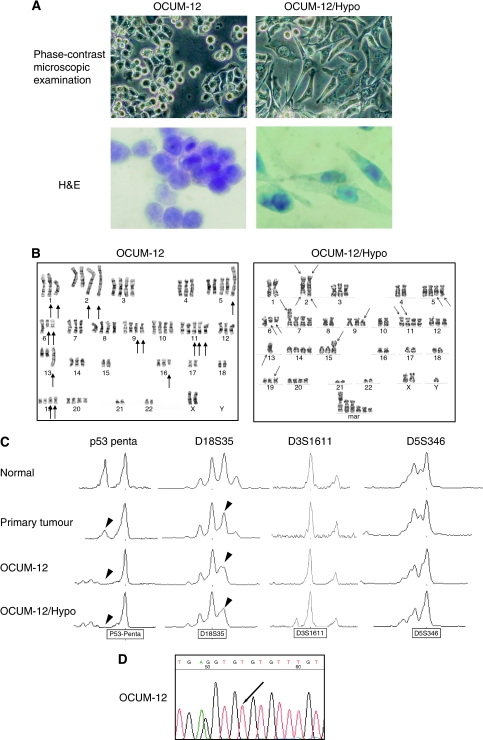
Morphology of OCUM-12 and OCUM-12/Hypo cells (**A**). OCUM-12 cells showed a round shape, whereas OCUM-12/Hypo cells were spindle-shaped and formed loose cell aggregates. G-banded karyotypes of OCUM-12 and OCUM-12/Hypo (**B**). The representative karyotype of OCUM-12 was XX, −X or −Y, add (1)(p36), del[1] (p32), add (4)(p11), +add (2)(q33), add(2)(q33), +3[2], del(3)(q26), +5, der(5)add(5)(p15), add(5)(q31), +6, del(6)(q13), del(6), +7, +8, +9, add(9)(p11), add(9), +10, +11, der(11)t(11;17)(p11;q11), der(11)t(11;17) × 2, −13, add(13)(q32), +14, add(15)(q24), add(16)(q11), −17, −18, +19, add(19)(p13) × 2, +20, −21, −22, +mar1, +mar2. The representative karyotype of OCUM-12/Hypo. XXY, −1, −2, der(2) add (2)(p23) add(2)(q33) × 2, −4, +5, del(5)(q31q33) × 2, del(6)(q13) × 2, +7, add(7)(p22), −8, del(9)(p13), −10, +11, add(11)(p11.2) × 2, −13, −13, add(13)(q34), +14, add(15)(p11.2), −16, −17, −18, add(19)(p13), +20, −21, −22, +5–7 mar. Arrows, breakpoints present. (**C**) Loss of heterozygosity analysis electropherograms using microsatellite markers. The primary gastric tumour, corresponding normal sample, OCUM-12, and OCUM-12/Hypo cells are shown by comparing the height and position of peaks. The primary tumour, OCUM-12, and OCUM-12/Hypo cells had LOH at the *p53* locus and *DCC/DPC4* locus, whereas no band shift was detected. The same size alleles at *D18S35*, *D3S1611*, and *D5S346* probes were found in normal epithelium, primary tumour, OCUM-12, and OCUM-12/Hypo cells, which shows that the OCUM-12 and OCUM-12/Hypo cell lines are derived from the same patient. *p53* sequencing analysis showed C>T transition (arrow) at codon 273 in exon 8 (**D**).

A hypoxia-resistant cell line, OCUM-12/Hypo, was successfully established from OCUM-12, a parent scirrhous gastric cancer cell line. The two cell lines show some morphologic and functional differences. We observed that OCUM-12 cells were round, whereas OCUM-12/Hypo cells were spindle shaped and showed epithelial-to-mesenchymal transition (EMT) with reduced intercellular adhesion (Figure 2A). The migratory ability of OCUM-12/Hypo cells (Supplementary Movie 1) was greater in comparison to that of OCUM-12 cells (Supplementary Movie 2). Both cell lines had irregular nuclei of variable size. The doubling times estimated from the growth curves of OCUM-12 and OCUM-12/Hypo were 13.4 and 24.9 h, respectively. The doubling time estimated from the growth curves of OCUM-12/Hypo was 20.0 h, when they returned to 20% O_2_. The levels of tumour-associated antigens CEA, CA19-9, and SPan-1 were detected at 98.0 ng ml^−1^, 5.8, and 6.0 U ml^−1^ in OCUM-12 cells and 68.7 ng ml^−1^, 22.3, and 7.5 U ml^−1^ in OCUM-12/Hypo cells. The AFP levels were within the normal range (<0.4 ng ml^−1^) in both cell lines.

### Chromosome analysis

The chromosome number varied between 71 and 78 (with a mode of 75) for OCUM-12, and ranged between 61 and 69 (with a mode of 67) for OCUM-12/Hypo. Of 50, 10 metaphase spreads were karyotyped. Figure 2B shows the major karyotypic features of OCUM-12 and OCUM-12/Hypo. The arrowheads indicate rearranged chromosomes. The chromosome markers common to the two cell lines were add(2)(q33), del(6)(q13), and add(19)(p13). Other structural abnormalities of OCUM-12 were add(1)(p36), del(1)(p32), add(4)(p11), del(3)(q26), der(5)add(5)(p15), add(5)(q31), add(9)(p11), der(11)t(11;17)(p11;q11), der(11)t(11;17), add(13)(q32), add(15)(q24), and add(16)(q11). Other structural abnormalities specific to OCUM-12/Hypo were add(2)(p23), del (5)(q31q33), add(7)(p22), del(9)(p13), add(11)(p11.2), add(13)(q34), and add(15)(p11.2).

### Genetic alterations

The primary tumour, OCUM-12, and OCUM-12/Hypo cells all showed an LOH at the *p53* locus and *DCC/DPC4* locus, whereas no band shift was detected (Figure 2C). The same size alleles at *D18S35*, *D3S1611*, and *D5S346* (which detect DNA polymorphisms) were found in normal epithelium, the primary gastric tumour, OCUM-12, and OCUM-12/Hypo cells, thus showing that the OCUM-12 and OCUM-12/Hypo cell lines are derived from the same patient. In both cell lines, *p53* sequencing analysis provided evidence of C>T transition (arrow) at codon 273 in exon 8, which leads to an amino-acid change from Arg>Cys (Figure 2D).

### Scirrhous gastric carcinoma model *in vivo*

**Figure 3:**
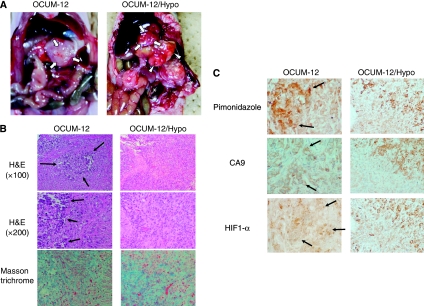
Orthotopic implantation model with OCUM-12 cells and OCUM-12/Hypo cells. (**A**) An orthotopic tumour in the stomach (arrows) was found 7 weeks after inoculation in both cell lines. Orthotopic implantation model with OCUM-12/Hypo cells yielded lymph node diseases (arrowheads) and peritoneal metastatic nodules (asterisks) 7 weeks after inoculation, whereas implantation with OCUM-12 cells developed few metastases. (**B**) Histologic findings of inoculated OCUM-12 and OCUM-12/Hypo tumours. Orthotopic tumours with OCUM-12 and OCUM-12/Hypo showed extensive fibrosis with the occasional presence of poorly differentiated adenocarcinoma cells. Masson's trichrome staining showed blue multiple fibrosis in the orthotopic tumours. Pimonidazole, HIF-1*α*, and CA9 staining were heterogeneously positive in OCUM-12 and OCUM-12/Hypo tumours (**C**).

At 7 weeks after the orthotopic inoculation of OCUM-12 or OCUM-12/Hypo cells in mice, gastric tumours were found in the middle wall of the stomach (Figure 3A). Tumours composed of OCUM-12/Hypo cells were associated with lymph node diseases and peritoneal disseminations (Figure 3A), whereas those derived from OCUM-12 cells showed few metastatic lesions. Orthotopic tumours derived from both OCUM-12 and OCUM-12/Hypo cells showed extensive fibrosis and the presence of poorly differentiated adenocarcinoma cells (Figure 3B), which resembled human scirrhous gastric carcinoma. Necrotic lesions were frequent in OCUM-12 tumours but were rarely found in OCUM-12/Hypo tumours (Figure 3B). The presence of hypoxia-associated endogenous proteins, such as HIF-1*α* and CA9, and pimonidazole, an exogenous marker, indicated that both tumour types had hypoxic loci (Figure 3C). The staining of pimonidazole, HIF-1*α*, and CA9 was heterogeneously positive in both the OCUM-12 and the OCUM-12/Hypo tumours (Figure 3C). The necrotic regions in the OCUM-12 tumours corresponded with HIF-1*α* or CA9-positive regions.

### Migratory and invasive ability

**Figure 4:**
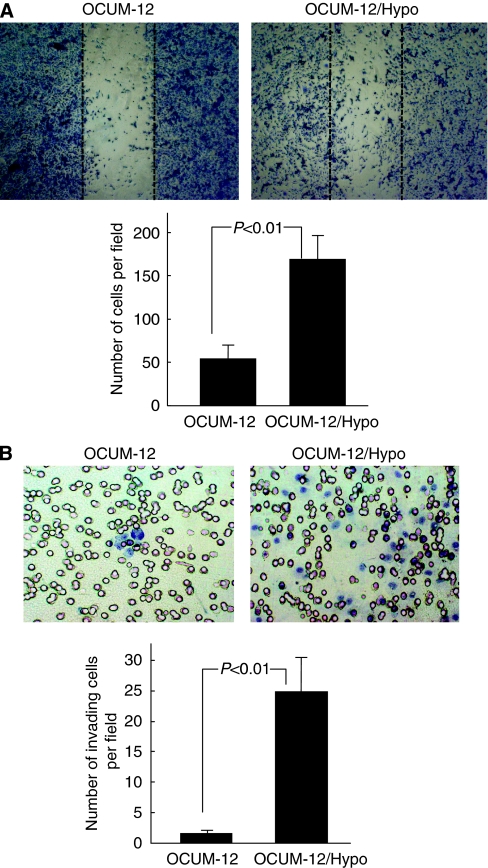
The motility of OCUM-12 cells and OCUM-12/Hypo cells *in vitro*. (**A**) Migration activity. Representative phase-contrast images of the wound-healing assay show the migrating cells in the wound margin. The number of migrating OCUM-12/Hypo cells was significantly (*P*<0.01) greater than that of OCUM-12 cells. Dotted line, wound margin. (**B**) Invasion activity. Representative phase-contrast images of the invasion assay show a higher number of invading OCUM-12/Hypo cells in comparison to OCUM-12 cells.

Figure 4A shows representative phase-contrast images of the *in vitro* wound-healing assay. The number of migrating OCUM-12/Hypo cells was about three times greater (*P*<0.01) than that of OCUM-12 cells. Figure 4B shows representative phase-contrast images of the *in vitro* invasion assay. The invasiveness of OCUM-12/Hypo cells was 18 times greater (*P*<0.01) than that of OCUM-12.

### Expression of adhesion molecules and matrix metalloproteinases

**Figure 5:**
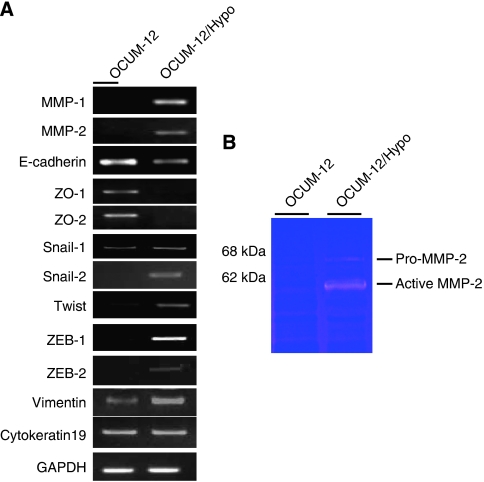
mRNA expression and Gelatinolytic activity. (**A**) mRNA expression. The mRNA expression levels of *vimentin*, *Snail-1*, *Slug/Snail-2*, *Twist*, *ZEB-1*, *ZEB-2*, *MMP-1*, and *MMP-2* were higher in OCUM-12/Hypo cells in comparison to the OCUM-12 cells. The expression levels of *E-cadherin*, *ZO1*, and *ZO2* in OCUM-12/Hypo cells were lower than those in OCUM-12 cells, whereas that of *cytokeratin 19* was not different. (**B**) Gelatinolytic activity. pro-MMP-2 (68 kDa) and active MMP-2 (62 kDa) were detected in OCUM-12/Hypo cells, but not in OCUM-12 cells.

The mRNA expression levels of cell–cell adhesion molecules, zonula occludens (*ZO)-1*, *ZO-2*, and *E-cadherin*, were lower in OCUM-12/Hypo cells in comparison to the OCUM-12 cells. However, the mRNA expression levels of *vimentin*, *Snail-1*, *Slug/Snail-2*, *Twist*, *ZEB-1*, *ZEB-2*, *MMP-1*, and *MMP-2* were higher in OCUM-12/Hypo cells in comparison to the OCUM-12 cells (Figure 5A). The mRNA expression level of cytokeratin 19 was not different between the two cell lines. Gelatinolytic activities corresponding to pro-MMP-2 (68 kDa) and the active form of MMP-2 (62 kDa) were detected in OCUM-12/Hypo cells but not in OCUM-12 cells (Figure 5B).

### Apoptosis

**Figure 6:**
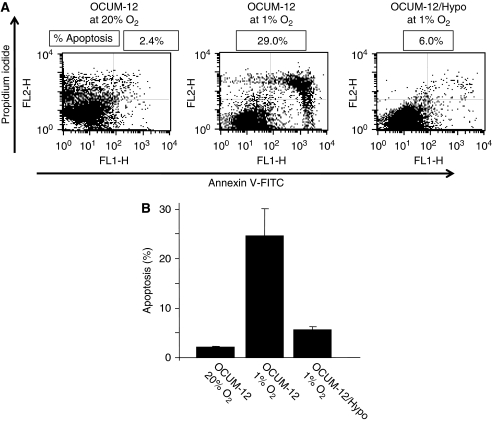
Apoptosis induction by hypoxia. (**A**) The typical examples of flow cytometric analysis. (**B**) In OCUM-12 cells, hypoxia induced apoptosis at a rate of 24.7%, whereas apoptosis rate at 20% O_2_ was 2.1%. In contrast, the apoptosis rate of OCUM-12/Hypo was 5.6% at hypoxia. The result represents the mean of three independent experiments and the bars show the s.d.

The apoptosis rate of OCUM-12 cells at 20% O_2_ was 2.1%. The apoptosis rate of OCUM-12 cells at 1% O_2_ hypoxia was increased to 24.7%, whereas that of OCUM-12/Hypo was 5.6% at 1% O_2_ (Figure 6).

## Discussion

In this study, we established a new hypoxia-resistant cell line, OCUM-12/Hypo, that could grow in a 1% oxygen environment from OCUM-12, a scirrhous gastric cancer cell line. Although hypoxia is cytotoxic to OCUM-12 cells, some cancer cells acquire characteristics that allow them to survive and grow in hypoxia (1% oxygen). To the best of our knowledge, no other hypoxia-resistant cell line has been reported. This is the first study to report the establishment and characterization of a stable cancer cell line that grows at low oxygen level. Because diffuse-type gastric cancer shows less angiogenesis than intestinal-type carcinoma (Takahashi *et al*, 1996), this cell type might more useful for studying the conditions of chronic hypoxia *in vivo* than intestinal-type cells. In this study, HIF-1*α* and CA9 staining of biopsy specimens revealed that hypoxic loci existed in the primary diffuse-type gastric tumour from which the OCUM-12 cell line was derived. The hypoxia-resistant cells might be present in the parent OCUM-12 cells, and the OCUM-12/Hypo cells may have been selected *in vitro* by continuous exposure to hypoxia. The doubling time estimated from the growth curves of OCUM-12/Hypo cells was not different in 20% oxygen and in 1% oxygen. The characteristic phenotypes of OCUM-12/Hypo cells did not change when they were re-incubated in 20% oxygen (data not shown), which indicates that the acquired hypoxia resistance of OCUM-12/Hypo cells is irreversible and OCUM-12/Hypo might be established as a clone. Moreover, we attempted and subsequently failed to establish a hypoxia-resistant cell line from other gastric cancer cell lines, including NU-GC-4 (Nomura *et al*, 2001), MKN7 (Motoyama *et al*, 1986), and MKN45 (Motoyama *et al*, 1986). The OCUM-12/Hypo cells were exposed to 20% O_2_ when passaged. However, the serial passages were carried out quickly in a few minutes, and OCUM-12/Hypo cells were returned to 1% O_2_ immediately. Moen *et al* (2009) indicated that hyperoxia treatment for 90 min might cause the mesenchymal-to-epithelial transition ‘switches’ *in vivo*. In contrast, no morphologic change was found during the serial passages, because our serial passages were carried out for only a few minutes.

Microsatellite markers are valuable genetic markers for individual identification because they could detect DNA polymorphisms. The same size alleles at *D18S35*, *D3S1611*, and *D5S346* probes were found in normal epithelium, primary gastric tumour, OCUM-12, and OCUM-12/Hypo cells, which shows that the OCUM-12 and OCUM-12/Hypo cell lines are derived from the same patient.

We orthotopically injected OCUM-12 or OCUM-12/Hypo cells into a murine stomach. Histologic findings of orthotopic tumours by OCUM-12 and OCUM-12/Hypo showed a poorly differentiated adenocarcinoma with extensive fibrosis, which resembled human scirrhous gastric cancer. Immunohistochemical analysis based on hypoxia-associated endogenous proteins such as HIF-1, CA9, or pimonidazole, an exogenous marker that is preferentially trapped in hypoxic cells, can yield tumour hypoxia information with direct comparison to histologic findings (Takahashi *et al*, 1996). In this study, necrotic lesions were detected in hypoxic areas of OCUM-12 tumours but were rarely found in OCUM-12/Hypo tumours, whereas pimonidazole, CA9, and HIF-1*α* staining revealed both areas to be hypoxic. Pimonidazole is positive at the hypoxic site of tumour cells under conditions of partial oxygen tension <10 mm Hg (Takahashi *et al*, 1996; Mayer *et al*, 2008). Orthotopic tumours derived from either OCUM-12 or OCUM-12/Hypo cells have heterogeneously hypoxic regions under and over 10 mm Hg O_2_. These findings indicate that OCUM-12/Hypo cells have acquired resistance to hypoxia and can survive in conditions with an oxygen tension <10 mm Hg. Further studies are therefore necessary to clarify the mechanisms responsible for survival in an environment with a partial oxygen tension <10 mm Hg.

It has been reported that tumour hypoxia is associated with more aggressive tumour phenotypes, which may lead to a poorer prognosis for patients (Imai *et al*, 2003; Driessen *et al*, 2006; Mayer *et al*, 2008). Our hypoxia models using OCUM-12/Hypo cells showed frequent lymph node metastasis. Previous studies have reported that the phenotypes of metastatic cancer cells are associated with migration ability and EMT, which is characterized by the loss of epithelial cell markers of cell–cell adhesion molecules (Imai *et al*, 2003; Jung *et al*, 2006; Revenu and Gilmour, 2009). OCUM-12/Hypo cells are spindle shaped and show EMT, whereas most OCUM-12 cells are round. The Supplementary Movies show that OCUM-12/Hypo cells display increased pseudopod formation, which is a prominent feature of actively motile cells. The expression levels of the cell–cell adhesion molecules *ZO-1*, *ZO-2*, and *E-cadherin* decreased in OCUM-12/Hypo cells. These findings suggest that hypoxia-induced EMT of OCUM-12/Hypo cells might be mediated through the regulation of cell–cell adhesion molecules. An important aspect of EMT is the loss of epithelial markers such as cytokeratin and E-cadherin, and the upregulation of mesenchymal markers such as vimentin. In this study, the mRNA expression level of *E-cadherin* was decreased and that of *vimentin* was increased in hypoxia-resistant cells, whereas that of *cytokeratin 19* was not different. Kim *et al* (2009b) reported that E-cadherin loss and the expression of vimentin was found to be significantly associated with an unfavourable prognosis of patients with gastric cancer, but no significant difference in patient outcome was found regarding cytokeratin expression status. Among epithelial and mesenchymal proteins, E-cadherin and Vimentin expression might be linked to a more aggressive status in gastric carcinoma, but cytokeratin expression might not be correlated with malignant phenotype. The mRNA expression levels of *Snail-1*, *Slug/Snail-2*, *Twist*, *ZEB-1*, *ZEB-2* were higher in OCUM-12/Hypo cells in comparison with the OCUM-12 cells. Several transcription factors have been associated with the repression of E-cadherin, including the Snail-1, Slug/Snail-2, Twist, ZEB-1, and ZEB-2 (Imai *et al*, 2003; Moen *et al*, 2009). These findings may show that hypoxia induces downregulation of E-cadherin in gastric carcinoma cells, through upregulation of the transcriptional repressor Snail, Twist, and ZEB families. Our observations indicate that OCUM-12/Hypo cells have a greater degree of invasive ability than OCUM-12 cells. The expression level of type IV collagenase (MMP-2 and MMP-9) correlates to carcinomatous malignancy (Sato *et al*, 2005). ProMMP-2 and active MMP-2 were detected in OCUM-12/Hypo cells, but not in OCUM-12 cells. Reverse transcription–PCR revealed that MMP-2 mRNA was expressed in OCUM-12/Hypo cells, but not in OCUM-12 cells. A wound-healing assay also showed that the migration capacity of OCUM-12/Hypo cells was much greater than that of OCUM-12 cells. These findings suggest that a highly invasive ability, MMP production, and a migratory ability (which are all enhanced by hypoxic conditions) may underlie the increased metastatic potential of hypoxic cancer cells.

Most gastric cancer cells produce various tumour-associated antigens (Yashiro *et al*, 1995), which function as adhesion molecules. Both cell lines produced the tumour-associated antigens CEA and CA19-9 at high concentrations, although the level of CEA was higher in OCUM-12 cells than in OCUM-12/Hypo cells. Because CEA is reported to be a cell-to-cell adhesion molecule (Benchimol *et al*, 1989), reduced CEA expression may facilitate EMT in cells and may increase the invasive ability. The CA19-9 production level was higher in OCUM-12/Hypo cells than in OCUM-12 cells. Some studies have reported that the overexpression of CA19-9 is responsible for adhesion to human umbilical vein endothelial cells in liver metastatic lesions (Takada *et al*, 1993). This ability to adhere to endothelial cells might enable cancer cells to survive in hypoxic environments. The above-mentioned findings suggest that differential expression of CEA and CA19-9 in hypoxic cells might be associated with the development of malignant potential in conditions of hypoxia.

Chromosome analysis showed many chromosome aberrations. One chromosome marker add(19)(p13) was common to both cell lines. Some hypoxia-associated genes, including follistatin like 3 (*FSTL3*), *CA9*, mitogen-activated protein kinase organizer 1 (*MORG1*), and angiopoietin-like 4 (*ANGPTL4*), are located near 9p13 (Hayette *et al*, 1998; Biron-Shental *et al*, 2008). The upregulation of *FSTL3* mRNA has previously been observed as a response to hypoxia in human trophoblasts, thus suggesting a role for HIF1-*α* (Biron-Shental *et al*, 2008). Carbonic anhydrases are a large family of zinc metalloenzymes that catalyse the reversible hydration of carbon dioxide. They participate in a variety of biologic processes, including respiration, acid-base balance, and bone resorption. *CA9* is expressed in epithelial cells with high proliferative capacities, thus supporting its role in cell proliferation. This CA has been implicated in the regulation of the microenvironmental pH by conversion of carbon dioxide into bicarbonate and hydrogen. Carbon dioxide and lactate, produced during anaerobic glycolysis in tumours, are important sources of acidity, which promotes tumour growth and metastasis. Based on these features, *CA9* is not only a tumour marker but also appears to be directly involved in the oncogenesis of different types of tumours. *MORG1* enhances ERK activation at low concentrations, whereas high concentrations lead to the inhibition of ERK activation. *ANGPTL4* is a member of the angiopoietin/angiopoietin-like gene family and encodes a glycosylated, secreted protein with a fibrinogen C-terminal domain. This gene is induced under hypoxic conditions in endothelial cells and is the target of peroxisome proliferation activators. The encoded protein is a serum hormone directly involved in regulating glucose homeostasis, lipid metabolism, and insulin sensitivity, and it also functions as an apoptosis survival factor for vascular endothelial cells. The encoded protein may have a role in several cancers and has been shown to repress the metastatic process by inhibiting vascular activity as well as tumour cell motility and invasiveness.

Structural abnormalities of OCUM-12/Hypo include add(2)(p23), where human xanthine dehydrogenase is located (Ichida *et al*, 1993). Xanthine dehydrogenase belongs to the group of molybdenum-containing hydroxylases involved in the oxidative metabolism of purines. The enzyme complex exists in separate but inter-convertible forms, namely xanthine dehydrogenase and xanthine oxidase, which generate reactive oxygen species, a causative factor in ischemia/reperfusion injury and other pathologic states and diseases. Xanthine oxidoreductase expression in gastric cancer may be a new marker for more aggressive gastric cancer biology (Linder *et al*, 2006).

In the most hypoxic regions of tumours, p53 is stabilized and induces apoptosis. Recent studies have suggested that mutations in p53, a tumour suppressor gene, may be important in regulating the adaptive response of tumour cells to hypoxia by enhancing their survival and the release of pro-angiogenic factors (Kieser *et al*, 1994). It has even been suggested that the more malignant clones harbouring genetic defects such as mutations in p53 might be selected by hypoxia for the survival (Royds *et al*, 1998). In both OCUM-12 and OCUM-12/Hypo cells, *p53* mutation in exon 8 and LOH at the *p53* locus were detected. A typical sequence for the loss of tumour suppressor genes in a neoplasm is an inactivating mutation at one allele, followed by LOH affecting the other allele. These findings suggest that both cell lines show a p53 function loss because of p53 mutation in one allele and LOH at the other allele. Our data indicate that loss of p53 function might have important role in tumour cell growth under hypoxic conditions.

In conclusion, we have established two new scirrhous gastric cancer cell lines, designated OCUM-12 and OCUM-12/Hypo. OCUM-12/Hypo cells can survive and grow in an environment of chronic hypoxia and show more frequent EMT, increased migratory and invasive activities, and higher metastatic potential than OCUM-12 cells. Our findings suggest that chronic hypoxia enhances the malignant phenotype of cancer cells. These two cell lines should be beneficial for the elucidation of the disease progression of scirrhous gastric cancer in chronic hypoxic conditions *in vitro* and *in vivo*.

## Figures and Tables

**Table 1:** Primer sequence

**Primer name**	**Primer sequence (5′–3′)**
MMP-1-S	TCGTGAGAATGTCTTCCCATT
MMP-1-AS	TCTTGGATTGATTTGAGATAAGTGAAATC
	
MMP-2-S	CGGCCGCAGTGACGGA
MMP-2-AS	CATCCTGGGACAGACGGAAG
	
ZO-1-S	CGGTCCTCTGAGCCTGTAAG
ZO-1-AS	GGATCTACATGCGACGACAA
	
ZO-2-S	GCCAAAACCCAGAACAAAGA
ZO-2-AS	ACTGCTCTCTCCCACCTCCT
	
Snail-1-S	GGGAAT TCTATGCCGCGCTCTTTCCTCGTC
Snail-1-AS	GGGGATCCTCAGCGGGGACATCCTGAGCAG
	
Snail-2-S	CTTTTTCTTGCCCTCACTGC
Snail-2-AS	ACAGCAGCCAGATTCCTCAT
	
ZEB-1-S	TGCACTGAGTGTGGAAAAGC
ZEB-1-AS	TGGTGATGCTGAAAGAGACG
	
ZEB-2-S	CGCTTGACATCACTGAAGGA
ZEB-2-AS	CTTGCCACACTCTGTGCATT
	
TWIST-S	GGAGTCCGCAGTCTTACGAG
TWIST-AS	TCTGGAGGACCTGGTAGAGG
	
Vimentin-S	AAGGGGACCAACGAGTCTCT
Vimentin-AS	TGACATTCAGCAGGTCTTGG
	
E-cadherin-S	TGATGCCCCCAATACCCCAG
E-cadherin-AS	CTGTGGAGGTGGTGAGAGAG
	
Cytokeratin 19-S	CCCGCGACTACAGCCACTA
Cytokeratin 19-AS	GCTCATGCGCAGAGCCTGTT
	
GAPDH-S	ACCTGACCTGCCGTCTAGAA
GAPDH-AS	TCCACCACCCTGTTGCTGTA

## References

[bib1] Albini A, Iwamoto Y, Kleinman HK, Martin GR, Aaronson SA, Kozlowski JM, McEwan RN (1987) A rapid *in vitro* assay for quantitating the invasive potential of tumor cells. Cancer Res 47: 3239–3245 2438036

[bib2] Benchimol S, Fuks A, Jothy S, Beauchemin N, Shirota K, Stanners CP (1989) Carcinoembryonic antigen, a human tumor marker, functions as an intercellular adhesion molecule. Cell 57: 327–334 2702691 10.1016/0092-8674(89)90970-7

[bib3] Biron-Shental T, Schaiff WT, Rimon E, Shim TL, Nelson DM, Sadovsky Y (2008) Hypoxia enhances the expression of follistatin-like 3 in term human trophoblasts. Placenta 29: 51–57 17959243 10.1016/j.placenta.2007.09.001 PMC2237886

[bib4] Borensztajn K, Stiekema J, Nijmeijer S, Reitsma PH, Peppelenbosch MP, Spek CA (2008) Factor Xa stimulates proinflammatory and profibrotic responses in fibroblasts via protease-activated receptor-2 activation. Am J Pathol 172: 309–320 18202198 10.2353/ajpath.2008.070347 PMC2312363

[bib5] Brown JM (1979) Evidence for acutely hypoxic cells in mouse tumours, and a possible mechanism of reoxygenation. Br J Radiol 52: 650–656 486895 10.1259/0007-1285-52-620-650

[bib6] Driessen A, Landuyt W, Pastorekova S, Moons J, Goethals L, Haustermans K, Nafteux P, Penninckx F, Geboes K, Lerut T, Ectors N (2006) Expression of carbonic anhydrase IX (CA IX), a hypoxia-related protein, rather than vascular-endothelial growth factor (VEGF), a pro-angiogenic factor, correlates with an extremely poor prognosis in esophageal and gastric adenocarcinomas. Ann Surg 243: 334–340 16495697 10.1097/01.sla.0000201452.09591.f3 PMC1448952

[bib7] Fujiwara I, Yashiro M, Kubo N, Maeda K, Hirakawa K (2008) Ulcerative colitis-associated colorectal cancer is frequently associated with the microsatellite instability pathway. Dis Colon Rectum 51: 1387–1394 18546042 10.1007/s10350-008-9212-9

[bib8] Furuta E, Pai SK, Zhan R, Bandyopadhyay S, Watabe M, Mo YY, Hirota S, Hosobe S, Tsukada T, Miura K, Kamada S, Saito K, Iiizumi M, Liu W, Ericsson J, Watabe K (2008) Fatty acid synthase gene is up-regulated by hypoxia via activation of Akt and sterol regulatory element binding protein-1. Cancer Res 68: 1003–1011 18281474 10.1158/0008-5472.CAN-07-2489

[bib9] Harris JW, Shrieve DC (1979) Effects of adriamycin and X-rays on euoxic and hypoxic EMT-6 cells *in vitro*. Int J Radiat Oncol Biol Phys 5: 1245–1248 528271 10.1016/0360-3016(79)90647-3

[bib10] Hayette S, Gadoux M, Martel S, Bertrand S, Tigaud I, Magaud JP, Rimokh R (1998) *FLRG* (follistatin-related gene), a new target of chromosomal rearrangement in malignant blood disorders. Oncogene 16: 2949–2954 9671416 10.1038/sj.onc.1201807

[bib11] Hockel M, Schlenger K, Aral B, Mitze M, Schaffer U, Vaupel P (1996) Association between tumor hypoxia and malignant progression in advanced cancer of the uterine cervix. Cancer Res 56: 4509–4515 8813149

[bib12] Hockel M, Vaupel P (2001) Tumor hypoxia: definitions and current clinical, biologic, and molecular aspects. J Natl Cancer Inst 93: 266–276 11181773 10.1093/jnci/93.4.266

[bib13] Ichida K, Amaya Y, Noda K, Minoshima S, Hosoya T, Sakai O, Shimizu N, Nishino T (1993) Cloning of the cDNA encoding human xanthine dehydrogenase (oxidase): structural analysis of the protein and chromosomal location of the gene. Gene 133: 279–284 8224915 10.1016/0378-1119(93)90652-j

[bib14] Imai T, Horiuchi A, Wang C, Oka K, Ohira S, Nikaido T, Konishi I (2003) Hypoxia attenuates the expression of E-cadherin via up-regulation of SNAIL in ovarian carcinoma cells. Am J Pathol 163: 1437–1447 14507651 10.1016/S0002-9440(10)63501-8 PMC1868286

[bib15] Japanese Gastric Cancer Association (1998) Japanese classification of gastric carcinoma – 2nd English edition. Gastric Cancer 1: 10–24 11957040 10.1007/s101209800016

[bib16] Jung AC, Ribeiro C, Michaut L, Certa U, Affolter M (2006) Polychaetoid/ZO-1 is required for cell specification and rearrangement during *Drosophila* tracheal morphogenesis. Curr Biol 16: 1224–1231 16782014 10.1016/j.cub.2006.04.048

[bib17] Kieser A, Weich HA, Brandner G, Marme D, Kolch W (1994) Mutant p53 potentiates protein kinase C induction of vascular endothelial growth factor expression. Oncogene 9: 963–969 8108142

[bib18] Kim JY, Kim YJ, Lee S, Park JH (2009a) The critical role of ERK in death resistance and invasiveness of hypoxia-selected glioblastoma cells. BMC Cancer 9: 27 19161638 10.1186/1471-2407-9-27 PMC2645423

[bib19] Kim MA, Lee HS, Lee HE, Kim JH, Yang HK, Kim WH (2009b) Prognostic importance of epithelial–mesenchymal transition-related protein expression in gastric carcinoma. Histopathology 54: 442–451 19309396 10.1111/j.1365-2559.2009.03247.x

[bib20] Linder N, Haglund C, Lundin M, Nordling S, Ristimaki A, Kokkola A, Mrena J, Wiksten JP, Lundin J (2006) Decreased xanthine oxidoreductase is a predictor of poor prognosis in early-stage gastric cancer. J Clin Pathol 59: 965–971 16935971 10.1136/jcp.2005.032524 PMC1860491

[bib21] Ludwig C (1984) Drug resistance of hypoxic tumour cells *in vitro*. Cancer Treat Rev 11(Suppl A): 173–178 6428739 10.1016/0305-7372(84)90057-4

[bib22] Mayer A, Hockel M, Wree A, Leo C, Horn LC, Vaupel P (2008) Lack of hypoxic response in uterine leiomyomas despite severe tissue hypoxia. Cancer Res 68: 4719–4726 18559518 10.1158/0008-5472.CAN-07-6339

[bib23] Moen I, Oyan AM, Kalland KH, Tronstad KJ, Akslen LA, Chekenya M, Sakariassen PO, Reed RK, Stuhr LE (2009) Hyperoxic treatment induces mesenchymal-to-epithelial transition in a rat adenocarcinoma model. PLoS One 4: e6381 19636430 10.1371/journal.pone.0006381 PMC2712688

[bib24] Motoyama T, Hojo H, Watanabe H (1986) Comparison of seven cell lines derived from human gastric carcinomas. Acta Pathol Jpn 36: 65–83 3962675 10.1111/j.1440-1827.1986.tb01461.x

[bib25] Nomura H, Nishimori H, Yasoshima T, Hata F, Sogahata K, Tanaka H, Nakajima F, Ikeda S, Kamiguchi K, Isomura H, Sato N, Denno R, Hirata K (2001) A novel experimental mouse model of peritoneal dissemination of human gastric cancer cells: analysis of the mechanism of peritoneal dissemination using cDNA macroarrays. Jpn J Cancer Res 92: 748–754 11473725 10.1111/j.1349-7006.2001.tb01157.x PMC5926777

[bib26] Revenu C, Gilmour D (2009) EMT 2.0: shaping epithelia through collective migration. Curr Opin Genet Dev 19: 338–342 19464162 10.1016/j.gde.2009.04.007

[bib27] Royds JA, Dower SK, Qwarnstrom EE, Lewis CE (1998) Response of tumour cells to hypoxia: role of p53 and NFκB. Mol Pathol 51: 55–61 9713587 10.1136/mp.51.2.55 PMC395611

[bib28] Sato H, Takino T, Miyamori H (2005) Roles of membrane-type matrix metalloproteinase-1 in tumor invasion and metastasis. Cancer Sci 96: 212–217 15819718 10.1111/j.1349-7006.2005.00039.x PMC11158816

[bib29] Seabright M (1971) A rapid banding technique for human chromosomes. Lancet 2: 971–972 10.1016/s0140-6736(71)90287-x 4107917

[bib30] Semenza GL (2002) HIF-1 and tumor progression: pathophysiology and therapeutics. Trends Mol Med 8: S62–S67 11927290 10.1016/s1471-4914(02)02317-1

[bib31] Song MS, Park YK, Lee JH, Park K (2001) Induction of glucose-regulated protein 78 by chronic hypoxia in human gastric tumor cells through a protein kinase C-epsilon/ERK/AP-1 signaling cascade. Cancer Res 61: 8322–8330 11719466

[bib32] Subarsky P, Hill RP (2003) The hypoxic tumour microenvironment and metastatic progression. Clin Exp Metastasis 20: 237–250 12741682 10.1023/a:1022939318102

[bib33] Tahara E (1990) Growth factors and oncogenes in human gastrointestinal carcinomas. J Cancer Res Clin Oncol 116: 121–131 2157713 10.1007/BF01612665 PMC12201737

[bib34] Takada A, Ohmori K, Yoneda T, Tsuyuoka K, Hasegawa A, Kiso M, Kannagi R (1993) Contribution of carbohydrate antigens sialyl Lewis A and sialyl Lewis X to adhesion of human cancer cells to vascular endothelium. Cancer Res 53: 354–361 7678075

[bib35] Takahashi Y, Cleary KR, Mai M, Kitadai Y, Bucana CD, Ellis LM (1996) Significance of vessel count and vascular endothelial growth factor and its receptor (KDR) in intestinal-type gastric cancer. Clin Cancer Res 2: 1679–1684 9816116

[bib36] Takemura S, Yashiro M, Sunami T, Tendo M, Hirakawa K (2004) Novel models for human scirrhous gastric carcinoma *in vivo*. Cancer Sci 95: 893–900 15546507 10.1111/j.1349-7006.2004.tb02199.x PMC11159367

[bib37] Vaupel P (2004) The role of hypoxia-induced factors in tumor progression. Oncologist 9(Suppl 5): 10–17 10.1634/theoncologist.9-90005-10 15591418

[bib38] Vaupel P, Fortmeyer HP, Runkel S, Kallinowski F (1987) Blood flow, oxygen consumption, and tissue oxygenation of human breast cancer xenografts in nude rats. Cancer Res 47: 3496–3503 3581084

[bib39] Vaupel P, Mayer A, Briest S, Hockel M (2003) Oxygenation gain factor: a novel parameter characterizing the association between hemoglobin level and the oxygenation status of breast cancers. Cancer Res 63: 7634–7637 14633681

[bib40] Yashiro M, Chung YS, Nishimura S, Inoue T, Sowa M (1995) Establishment of two new scirrhous gastric cancer cell lines: analysis of factors associated with disseminated metastasis. Br J Cancer 72: 1200–1210 7577468 10.1038/bjc.1995.486 PMC2033934

[bib41] Yashiro M, Chung YS, Nishimura S, Inoue T, Sowa M (1996) Peritoneal metastatic model for human scirrhous gastric carcinoma in nude mice. Clin Exp Metastasis 14: 43–54 8521616 10.1007/BF00157685

