# Supplementary material for: Establishment and characterization of a new hypoxia-resistant cancer cell line, OCUM-12/Hypo, derived from a scirrhous gastric carcinoma
Source: Br J Cancer. 2010 Feb 9;102(5):898–907. doi: 10.1038/sj.bjc.6605543 (PMC2833244; doi:10.1038/sj.bjc.6605543)
Supplement: The Legends for Supplementary Movies [file 6605543x3.doc]

**The legends for supplementary movies.**

**Supplement movie 1. Real time living cell imaging of OCUM-12/Hypo cells.**

**Supplement movie 2. Real time living cell imaging of OCUM-12 cells.**

Real time living cell imaging of OCUM-12/Hypo and OCUM-12 cells was monitored by time-lapse video microscopy for 72 h at a 15 min interval. Real time monitoring showed that the migration ability of OCUM-12/Hypo cells (**Supplement movie 1**) was greater in comparison to that of OCUM-12 cells (**Supplement movie 2**). OCUM-12 cells were round, while OCUM-12/Hypo cells were spindle-shaped and displayed epithelial-to-mesenchymal transition (EMT) with reduced intercellular adhesion.
